# Classification of Packaged Vegetable Soybeans Based on Freshness by Metabolomics Combined with Convolutional Neural Networks

**DOI:** 10.3390/metabo15030145

**Published:** 2025-02-21

**Authors:** Yoshio Makino, Yuta Kurokawa, Kenji Kawai, Takashi Akihiro

**Affiliations:** 1 Graduate School of Agricultural and Life Sciences, The University of Tokyo, Tokyo 1138657, Japan; 2 BelleGreenWise Co., Ltd., Nagoya 4600007, Aichi, Japan; 3 Faculty of Life and Environmental Science, Shimane University, Matsue 6908504, Shimane, Japan

**Keywords:** artificial intelligence, cluster analysis, *Glycine max* (L.) Merrill, postharvest storage, modified atmosphere packaging

## Abstract

**Background/Objectives:** Effectiveness of modified atmosphere (MA) packaging for the preservation of the freshness of vegetable soybeans was confirmed by using metabolomics combined with convolutional neural networks (CNNs). **Methods:** Stored under a low O_2_, high CO_2_ environment, the vegetable soybeans’ freshness was tracked through changes in hue angle on the surface of the crops and metabolite levels compared to those stored under normoxia. **Results:** MA packaging slowed respiration and reduced pectin decomposition, succinic acid oxidation, and fatty acid consumption, all linked to freshness maintenance. Using 62 key metabolite concentrations as inputs, CNNs classified vegetable soybean freshness into seven categories with 92.9% accuracy, outperforming traditional linear discriminant analysis by 14.3%. **Conclusions:** These findings demonstrate MA packaging’s effectiveness in extending freshness of vegetable soybeans by monitoring specific metabolic changes. This will contribute to the advancement of research aimed at elucidating the relationship between freshness and metabolism in horticultural crops.

## 1. Introduction

Fruits and vegetables continue their vital activities while consuming the nutrients they possess even after harvesting, which causes deterioration in freshness such as discoloration and wilting, for example. Horticultural product deterioration is closely related to respiration rate, and it has been reported that products with rapid respiration have a shorter shelf life [1]. Since this suggests that suppression of respiration is effective for maintaining freshness, a method of reducing respiration rate through a method such as refrigeration to maintain freshness has been put into practice [2].

Various methods can be used to objectively assess the effectiveness of the freshness preservation method. Cia et al. [3] evaluated the freshness of persimmons (*Diospyros kaki* Thunb.) by measuring the hardness of the fruit flesh, which progressed more as the solubilization of pectin contributed to deterioration. Lipid peroxidation has been related to the senescence in broccoli (*Brassica oleracea* var. *italica*) buds [4] and cabbage (*Brassica oleracea* var. *capitata*) leaves [5]. The evaluation methods mentioned above are examples of research in which freshness is quantified with a single evaluation index. However, there are other reports in which plural indices are synthesized to quantify freshness. Makino and Amino [6] quantified the freshness of broccoli by synthesizing a single index from mass retention and green color retention rates by linear discriminant analysis (LDA). Li et al. [7] predicted the freshness of vegetable soybeans (*Glycine max* (L.) Merrill) by performing a partial least square regression analysis with 98 types of chlorophyll fluorescence parameters as input variables.

In recent years, metabolomics has been heavily used in plant studies [8]. Studies on horticultural crops with metabolomics continued to grow, and Chen et al. [9] reported that the breeding of vegetable soybeans changed the profile of metabolites. According to Sugimoto et al. [10], the metabolite profile in vegetable soybeans changes over time after harvest. This suggests that deterioration affects metabolite profiles. In addition, Pedreschi et al. [11] reported that metabolomics was effective for investigating the ripening mechanism of avocado fruit, while Hatoum et al. [12] used metabolomics to characterize the influence of the use of chemicals (calcium, potassium, and triazole fungicides) on primary metabolites in Braeburn apples (*Malus pumila* Mill. var. *domestica* (Borkh.) C.K. Schneid.). Pedreschi et al. [13] were able to identify the cause of core brown in Conference pears (*Pyrus communis* L.) by metabolic profiling under reduced O_2_ and elevated CO_2_ conditions. Syukri et al. [14] identified abscisic acid as a freshness indicator for soybean sprouts through metabolomics. Makino et al. [15] found that the concentrations of some functional food ingredients as niacin amid in vegetable soybeans were increased by storing under reduced O_2_ and elevated CO_2_ conditions at 25 °C through metabolomics with machine learning. Li et al. [16] revealed the correlation between the freshness of komatsuna and ten amino acids using NMR metabolomics.

In the current study, the freshness-preserving effect of modified atmosphere (MA) packaging was also quantitatively evaluated by metabolomics combined with deep learning, as an artificial intelligence (AI), and the results are reported. The objective of this study was to elucidate the freshness-preserving effects of MA packaging on vegetables based on the dynamic changes in metabolic components, as well as to confirm the potential for predicting storage duration as an indicator of freshness. In this study, we selected vegetable soybean, which is reported to exhibit significant freshness deterioration and freshness preservation effects by MA packaging [17], as the sample. This state-of-the-art technology may be demonstrated to be effective for evaluating freshness of vegetable soybeans.

## 2. Materials and Methods

### 2.1. Vegetable Materials

Soybeans, cv. “Yuagarimusume”, harvested on 27 June 2019 at a farm in Saitama prefecture in Japan (36.22 N, 139.22 E) 1 d before use in the following experiments, were directly transported to our laboratory (the University of Tokyo) under cool conditions (0 to 5 °C) for 1 d to minimize quality changes in sample quality as much as possible. This cultivar is one of the most widely consumed in Japan, making it an effective choice for generating the research findings.

### 2.2. Packaging Materials

The following two types of macro-perforated pouches constructed of oriented polypropylene (surface area: 0.08 m^2^, thickness: 0.04 mm; BelleGreenWise Co., Ltd., Nagoya, Japan) were used: those with six perforations (6 mm diameter) to simulate normoxic storage conditions and those with one perforation (6 mm diameter) to simulate a decreased O_2_/elevated CO_2_ modified environment. According to previous reports [13,15], the differences in the gas composition inside the pouches, attributable to the number of perforations, are thought to affect the dynamic changes in the metabolism of the contents (vegetable soybeans).

### 2.3. Measurement of In-Package Gas Composition, External Color, and Metabolite Concentrations

Five sets (250 g per set) of vegetable soybeans that had not been stored were prepared (beans and pods) and defined as 0-day-old samples (i.e., these represented stored soybeans on the first day of storage). In the current study, these samples before storage were used as controls. For the stored soybeans, 15 pouches (with 250 g of soybeans in each pouch) of both types (i.e., 6 and 1 perforations) were sealed and stored at 10 °C for 21 d to observe changes in freshness and metabolites at low temperatures. Following that, on 7, 14, and 21 d after the start of storage, five replicate pouches of each pouch type were randomly selected, and the atmosphere conditions (O_2_ and CO_2_ concentrations) in the pouches were determined using a gas analyzer (CheckMate 3; Dansensor A/S, Ringsted, Denmark).

According to the method described by Makino et al. [18], Commission Internationale de l’Éclairage (1976) L*a*b* color space values were measured using a computer vision system (CVS) (FMVU-13S2C-CS; Point Grey Research Inc., Richmond, BC, Canada). The hue angle (*H*, °) was then calculated to evaluate the green color as an index of the freshness of the vegetable soybeans [7] using the following equation:

(1) $$H={tan}^{-1}\left(\frac{b*}{a*}\right)\times \frac{180}{\pi },$$

Shortly after measuring the color space values, vegetable soybeans were milled in liquid nitrogen using a grind mixer (GM200; Verder Scientific GmbH & Co. KG, Haan, Germany). The resulting cold powder was dried using a freeze dryer and stored in a desiccator. Following that, 30 mg of dried powder was extracted to measure the concentrations of metabolites. Sample preparation for metabolite measurements followed the methods reported by Pongsuwan et al. [19] and Yokota et al. [20].

Metabolites were measured using a Shimadzu GC-MSQP2010 gas chromatograph–mass spectrometer (MS) (Shimadzu Co., Kyoto, Japan) and an Rtx-5MS column (30 m × 0.25 mm i.d., df = 0.25 μm; Restek Corporation, Bellefonte, PA, USA). The initial column temperature was maintained at 80 °C for 1 min and then increased to 320 °C at a rate of 15 °C min^−1^. The MS conditions were set at a scan range of 50–1000 *m*/*z*, ion source temperature 200 °C, interface temperature 250 °C, scan speed 5000 μs^−1^, and event time 0.2 s. Ribitol was used as an internal standard [21] to validate stability of the device.

Smart Metabolites Database ver. 2 (Shimadzu Co.) was used to detect peaks and calculate peak areas. We established retention indices (RIs) using n-alkanes. Metabolite identification was performed using this database. Among the 428 registered compounds, metabolites were automatically identified based on the following criteria:

1. Retention time within ±0.2 min of the database entry;
2. A spectral similarity score of ≥70% compared to the standard mass spectra in the database.

Furthermore, all automatically identified spectra were re-evaluated using similarity searches for confirmation.

The distribution of each specific metabolite concentration was normalized to the range of 0–1 using the following equation according to the method of Yokota et al. [20]:

(2) $$C_{s}=\frac{C_{i}-C_{min}}{C_{max}-C_{min}}$$

where *C* denotes the specific concentration of the compound, subscript *s* denotes the normalized value, subscript *i* denotes an arbitrary value, *max* denotes the maximum value, and *min* denotes the minimum value.

### 2.4. Statistical Analysis

All data were obtained from five different biological samples. Mean data for gas concentration and *H* were compared using Tukey’s honestly significant difference (HSD) test. Cluster analysis (Ward’s method) was conducted to visualize dynamic changes in normalized metabolite concentrations. Five replicates of each storage condition treatment were assigned to calibration and validation sets with a ratio of 3:2, to conduct the classification of samples into seven types of storage conditions (0 d; normoxia 7, 14, and 21 d; MA 7, 14, and 21 d). Each set of normalized metabolite concentrations quantified in a sample were tagged with a storage condition of the same sample. The samples were classified using deep learning, specifically convolutional neural networks (CNNs). Additionally, LDA was performed as a conventional method for classification, and the classification accuracy was compared between CNNs and LDA. Tukey’s HSD test, cluster analysis, and LDA were conducted using JMP ver. 14.2.0 software (SAS Institute Inc., Cary, NC, USA). CNNs were implemented using Sony Neural Network Console ver. 2.10 [22]. This is a tool for creating training and evaluating machine learning models. Users can create deep learning programs through visual operations without writing programming code.

## 3. Results and Discussion

### 3.1. Changes in In-Package Atmosphere over Time

**Figure 1:**
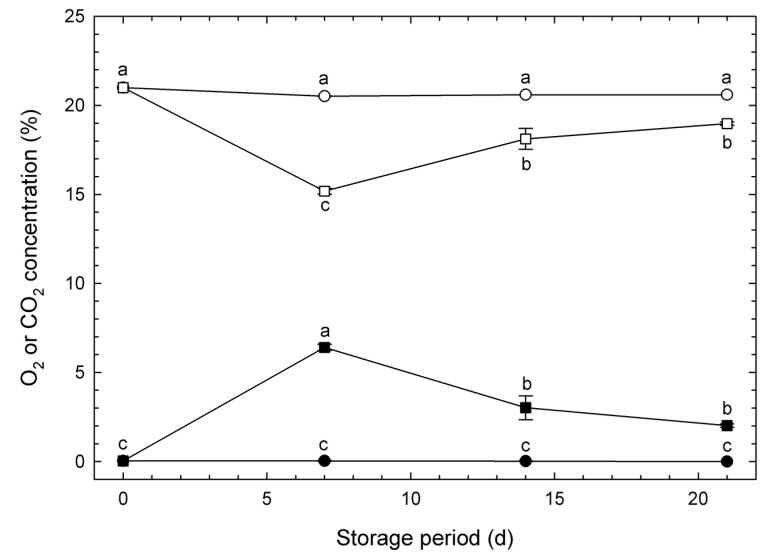
Temporal changes in O_2_ (unfilled symbols) and CO_2_ (filled symbols) concentrations inside oriented polypropylene pouches with perforations containing vegetable soybeans stored for 21 d at 10 °C. Symbols: Circles denote pouches with six perforations of 6 mm diameter (normoxia). Squares denote pouches with one perforation of 6 mm diameter (creating a reduced O_2_ and elevated CO_2_, modified atmosphere). Values are presented as the means ± SE of observations from five different biological samples. Symbols with the same letter over them, for the same type of gas, denote no significant difference at *p* < 0.05 using Tukey’s honestly significant difference test.

The concentrations of package gases during storage are shown in Figure 1. The concentrations of O_2_ and CO_2_ in pouches with six perforations were similar to those of ambient air (normoxia). Pouches with one perforation maintained O_2_ concentrations in the range of 15.0–18.9% (significantly lower than pouches with six perforations) during storage, whereas the CO_2_ concentrations ranged from 2.1 to 6.7% (significantly higher than pouches with six perforations) during storage. These values were determined to be appropriate for analyzing the effect of the atmosphere on the external color of stored vegetable soybeans as well as tracking the dynamic changes in their metabolites over time.

During the storage period (21 d), the mass loss of the vegetable soybeans was 25.0% in pouches with six perforations and 1.7% in pouches with one perforation, respectively.

### 3.2. Changes in Hue Angle on the Surface of Vegetable Soybeans over Time

**Figure 2:**
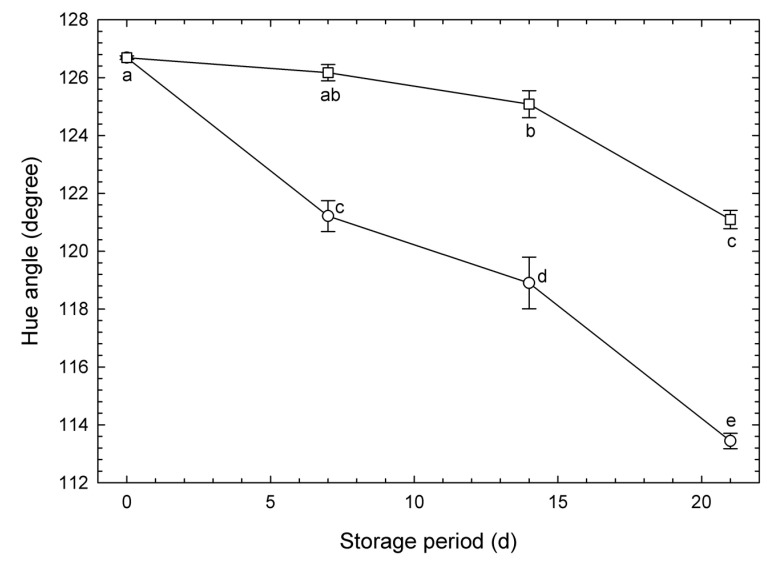
Temporal changes in hue angles of vegetable soybeans sealed in oriented polypropylene pouches with perforations stored for 21 d at 10 °C. Symbols: Circles denote pouches with six perforations of 6 mm diameter (normoxia). Squares denote pouches with one perforation of 6 mm diameter (creating a reduced O_2_ and elevated CO_2_, modified atmosphere). Values are presented as the means ± SE of observations from five different biological samples. Symbols with the same letter denote no significant differences at *p* < 0.05 using Tukey’s honestly significant difference test.

Under normoxia, the green color on the surface of vegetable soybeans (as measured by the hue angle) decreased continuously during storage in both types of pouches (Figure 2). Meanwhile, MA packaging retained a higher degree of greenness than those under normoxia from day 7 to 21. Reduced O_2_ and elevated CO_2_ concentrations in the surrounding atmosphere of stored vegetable soybeans were shown by Katsumi et al. [17] to be effective in maintaining the green color. As a result, sealing vegetable soybeans in pouches with a single perforation must have been effective in reducing CO_2_ loss and slowing O_2_ entry. A reduction in green color leads to a loss of salability and indicates a loss of nutritional quality [10]. Therefore, pouches with fewer perforations are better able to maintain green color in vegetable soybeans than those with more perforations.

### 3.3. Influence of Atmospheric Exposure During Storage on Metabolite Concentrations (Cluster Analysis)

**Figure 3:**
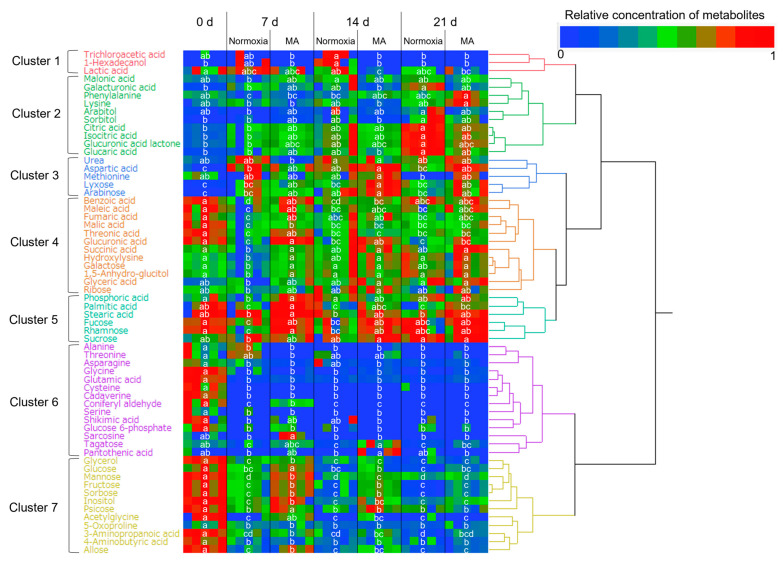
Cluster analysis (Ward’s method) of 62 types of metabolites (dry basis) in vegetable soybeans sealed in oriented polypropylene pouches with perforations and stored for 21 d at 10 °C. Normoxia (pouches with six perforations of 6 mm diameter) and MA (pouches with one perforation of 6 mm diameter, reduced O_2_, and elevated CO_2_). All data obtained from five different biological samples. Color cells with the same letter for the same metabolite denote no significant difference at *p* < 0.05 using Tukey’s honestly significant difference test.

Normalized concentrations of 62 types of metabolites, where the mean values were significantly affected by the storage conditions, were visualized as the heatmap combined with the dendrogram generated based on the results from cluster analysis in Figure 3.

The metabolites classified in Cluster 2 show significantly higher metabolite concentrations in normoxia on 21 d compared to the fresh samples (0 d). Galacturonic acid significantly increased in normoxia on 21 d compared to 0 d; however, it did not increase in the samples stored by MA packaging. As freshness of the produce decreases, insoluble pectin in the cell walls transforms into soluble pectin, which leads to the breakdown of cell structure and generation of galacturonic acid, a constituent monosaccharide [23]. It is presumed that the freshness deterioration was suppressed by MA packaging; hence, the significant increase in galacturonic acid was not observed. This result is consistent with previous research findings on tomato fruits [20].

Under the environmental conditions of normoxia, the free sugars (e.g., glucose) abundant in the fresh sample (0 d) were directed into the Krebs cycle via glycolytic metabolism, leading to significantly higher concentrations of cycle-associated metabolites—such as citric acid and isocitric acid—at 21 d compared to 0 d [24]. In contrast, under MA packaging, where metabolism was suppressed, no significant increase in the concentrations of metabolites related to the Krebs cycle was observed.

Succinic acid, a member of the tricarboxylic acid cycle, is oxidized by succinate dehydrogenase (SDH, EC1.3.5.1) with flavin adenine dinucleotide as a coenzyme. SDH functions as complex II in the respiratory chain and has been reported to be inhibited by CO_2_ [25]. According to the results in Figure 3, succinic acid was classified into Cluster 4, and at 7 d, its concentration in MA packaging samples was significantly higher than in normoxia. Referring to the results in Figure 1, the CO_2_ concentration inside the MA pouch reached its peak on 7 d. Therefore, it is suggested that succinic acid oxidation was inhibited due to the high CO_2_ concentration, leading to the observed increase in its concentration.

Palmitic acid and stearic acid, classified into Cluster 5, are major saturated fatty acids naturally present in the biological world. Originally, vegetable soybeans contain abundant lipids (66 g kg^−1^) [26]. The quantified palmitic acid and stearic acid in this study are considered to have been released from the triglycerides in vegetable soybeans. Fatty acids serve as an energy source for vegetables to sustain life activities after harvest. Therefore, it is presumed that these fatty acids were consumed as respiratory substrates. However, on 7 d, the concentration of these fatty acids in MA packaging samples was higher than in normoxia. This suggests that respiration was suppressed by MA packaging, potentially resulting in a higher remaining rate of fatty acids.

The metabolites classified into Cluster 6 exhibited high concentrations initially, with a tendency to decrease as the storage period progressed. In particular, free amino acids such as glutamic acid were present at high concentrations in the fresh samples (0 d) as protein degradation products, and during storage, they were utilized as respiratory substrates to sustain the metabolic activity of vegetable soybeans, which is thought to have resulted in a significant decrease in their concentrations [27].

Cluster 7 includes sugars such as glucose, fructose, and mannose. A common trend in concentration changes was observed: they were highest in fresh samples and decreased with the passage of storage time. This decrease is likely due to their consumption as an energy source for biological activities. Moreover, these sugars were higher in MA packaging samples than in normoxia on 7 d. This suggests that respiration was suppressed by MA packaging, leading to a higher remaining rate of sugars.

The compositional changes of environmental gases and the storage period dependence observed in metabolites in Figure 3 might be useful for freshness classification of vegetable soybeans using AI.

### 3.4. Prediction of the Freshness of Vegetable Soybeans by Deep Learning Based on Dynamic Changes in Metabolite Concentrations

The results of classifying samples based on the normalized metabolite concentrations shown in Figure 3 for each storage condition (environmental gas composition and storage period) are presented in Table 1.

**Figure 4:**
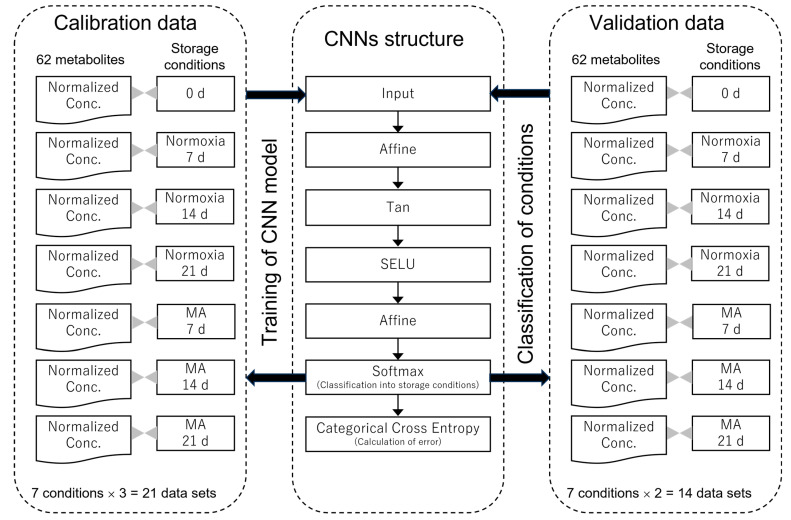
Execution conditions and architecture of the convolution neural networks (CNNs). Initial values in Sony Neural Network Console ver. 2.10 [22] were as follows: batch size 4, maximum number of epochs 100. Error calculated from categorical cross-entropy was 0.195. MA: modified atmosphere; Affine, Tan, SELU, Softmax, categorical cross-entropy: functions used in CNNs [22].

The results of LDA are shown in Table 1A. The accuracy was 11/14 × 100 = 78.6%. LDA, a conventional classification method for general data [28], was performed in this study for comparative purposes. In contrast, Table 1B presents the results of classification using CNNs. The accuracy was 13/14 × 100 = 92.9%, which was 14.3 points higher than the results from LDA. Deep learning including CNNs, a novel computational method for ANNs devised by Hinton in 2006 [29], has continued to evolve as cutting-edge AI technology, used in areas such as autonomous driving and chat tools. In this study, the execution conditions and architecture of the CNNs are illustrated in Figure 4. When applied to metabolomics as big data in the field of life sciences, deep learning demonstrated excellent performance, as shown in Table 1B.

The current study also presented measured data of *H* as a freshness indicator for vegetable soybean (Figure 2). As a result, no significant differences were observed between 0 d and 7 d under MA, between 7 d and 14 d under MA, or between 21 d under MA and 7 d under normoxia packaging, indicating equivalent freshness. However, when using CNNs, all classifications were correct except one individual on 7 d under normoxia. This suggests that normalized metabolite concentrations reflect the effects of storage atmosphere and storage period, serving as a detailed freshness evaluation indicator capable of observing the internal state of the sample.

As an example, comparing the normalized metabolite concentrations of MA 21 d and normoxia 7 d shown in Figure 3 revealed significant differences in the mean of two types in Cluster 2, six types in Cluster 4, and three types in Cluster 5. These data were considered helpful for distinguishing the storage environment history of the samples. In other words, the combination of metabolomics and CNNs proposed in this study allows for more detailed selection of freshness and quality compared to traditional freshness determination methods like *H*.

The present and previous reports combining metabolomics and machine learning in the postharvest field conducted in recent years are shown in Table 2. Various research results have been reported, but the novelty of this study lies in the integration of CNNs with metabolomics, specifically applied to postharvest research. As deep learning is a rapidly advancing technology, the findings of this study are expected to serve as a reference for future research targeting plants.

We consider the method proposed in this study, which involves determining the freshness of vegetables based on the dynamic changes in metabolites resulting from the MA packaging, to be a novel approach. In the future, the results reported in the present study will be useful in basic research on the freshness of vegetables.

## 4. Conclusions

This study investigated changes in the atmosphere within pouches and their effects on vegetable soybeans’ color (equivalent to freshness) and metabolites during storage. Pouches with varying perforations showed distinct O_2_ and CO_2_ concentrations, affecting the vegetable soybeans’ color retention and metabolic changes. Metabolite analysis revealed differences between normoxia and MA packaging, indicating effectiveness of MA packaging in preserving freshness. Notably, galacturonic acid, succinic acid, and fatty acids showed significant differences, possibly due to storage under low O_2_, high CO_2_, and respiration suppression in MA packaging. Deep learning, particularly CNNs, demonstrated superior accuracy (92.9%) in freshness prediction compared to LDA (78.6%), showcasing the potential of AI in metabolomics research. The integration of metabolomics and CNNs provides a detailed evaluation of freshness, surpassing traditional methods. This novel approach offers insights into plant metabolomics research and sets a precedent for future studies combining metabolomics and machine learning.

## Figures and Tables

**Table 1:** Prediction results on storage period and atmospheres (validation data set: two baches per condition) by machine learning.

**(A) Linear Discriminant Analysis**								
	**Pr**	**0 d**	**N-7 d**	**M-7 d**	**N-14 d**	**M-14 d**	**N-21 d**	**M-21 d**
**Ac**								
**0 d**		2	0	0	0	0	0	0
**N-7 d**		0	1	0	1	0	0	0
**M-7 d**		0	0	2	0	0	0	0
**N-14 d**		0	1	0	1	0	0	0
**M-14 d**		0	0	0	0	2	0	0
**N-21 d**		0	0	0	0	0	2	0
**M-21 d**		0	0	0	0	1	0	1
**(B) Convolutional Neural Networks**								
	**Pr**	**0 d**	**N-7 d**	**M-7 d**	**N-14 d**	**M-14 d**	**N-21 d**	**M-21 d**
**Ac**								
**0 d**		2	0	0	0	0	0	0
**N-7 d**		0	1	0	1	0	0	0
**M-7 d**		0	0	2	0	0	0	0
**N-14 d**		0	0	0	2	0	0	0
**M-14 d**		0	0	0	0	2	0	0
**N-21 d**		0	0	0	0	0	2	0
**M-21 d**		0	0	0	0	0	0	2

Ac: actual class; Pr: predicted class; black cells indicate misclassification; N: normoxia; M: modified atmosphere; 0−21 d: storage days; numbers in cells: batches of vegetable soybeans classified in the conditions.

**Table 2:** Present and previous reports on metabolomics with machine learning in postharvest technology.

Commodity	Variable	Data Analysis Method
Vegetable soybeans *	Storage atmosphere and period	CA, DL (CNNs)
Pineapple [30]	Ripeness stage	CA
Potato tuber [31]	Earliness of tuberization	PCA, CA
Vegetable soybeans [15]	Storage atmosphere and period	PCA, CA
Tomato [20]	Storage atmosphere and period	PCA
Soybean sprout [14]	Storage temperature	PCA-DA
Lettuce [32]	Genetic resource	CA
Mango [33]	Ripeness stage	PCA
Avocado [11]	Ripeness stage	PCA
Apple [12]	Storage period	PLS-DA
Vegetable soybeans [10]	Storage period	PCA, CA
Pear [13]	Low O_2_ stress	PLS-DA

* Present study. Abbreviations: CA: cluster analysis; CNNs: convolution neural networks; DL: deep learning; PCA: principal component analysis; PLS: partial least squares; DA: discriminant analysis.

## Data Availability

The raw data supporting the conclusions of this article will be made available by the authors on request.
